# Ultrasound and Three-Dimensional Image Reconstruction Angio-Computed Tomography Scan for Hybrid Surgical Tracheostomy in Patient with Abnormal Neck Vascularization

**DOI:** 10.1093/icvts/ivaf193

**Published:** 2025-08-21

**Authors:** Duilio Divisi, Luca Procaccini, Stefania De Sanctis, Chiara Angeletti

**Affiliations:** Department of Life, Health and Environmental Sciences, University of L’Aquila, Thoracic Surgery Unit, “Giuseppe Mazzini” Hospital of Teramo, 64100 Teramo, Italy; Interventional Radiology Unit, “Giuseppe Mazzini” Hospital of Teramo, 64100 Teramo, Italy; Department of Life, Health and Environmental Sciences, University of L’Aquila, Thoracic Surgery Unit, “Giuseppe Mazzini” Hospital of Teramo, 64100 Teramo, Italy; Emergency Department, Anesthesiology, Intensive Care and Pain Medicine, “Giuseppe Mazzini” Hospital of Teramo, 64100 Teramo, Italy

**Keywords:** surgical tracheostomy, Ciaglia Blue Rhino tracheostomy, ultrasound, three-dimensional angio-CT

## Abstract

Percutaneous dilatational tracheostomy is a common and safe ICU procedure. However, neck anatomy variations can cause bleeding. Pre-procedural ultrasound should be carried out to detect abnormalities in neck structures. A 3D image reconstruction angio-computed tomography scan of the epiaortic vessels should be performed for precise planning of actions. We describe a hybrid tracheostomy technique required following an anomalous vascularization of the neck discovered preoperatively by echography and imaging method.

## INTRODUCTION

Minimally invasive tracheostomy is considered a good alternative to the open surgical technique because it is easy and able to be performed quickly at the bedside.^1^ The method of Ciaglia consists of the introduction of the tracheostomy tube from an external to internal direction using multiple dilators of increasing calibre. To make the safer, faster, and easier percutaneous approach, Ciaglia modified the original technique using a conical and curved dilator marked beyond the 38-F linear reference (CBR: Ciaglia Blue Rhino).^2^ Preoperative ultrasound (US) and 3D images, angio-computed tomography scan of the neck, identifying early the abnormal vascular structures, can prevent fatal haemorrhage or difficult-to-manage bleeding during or after percutaneous dilatational tracheostomy (PDT).^3^^,^^4^ We experienced a surgical and percutaneous tracheostomy technique for an anomalous paratracheal venous vessel.

## CASE PRESENTATION

**Figure 1.**
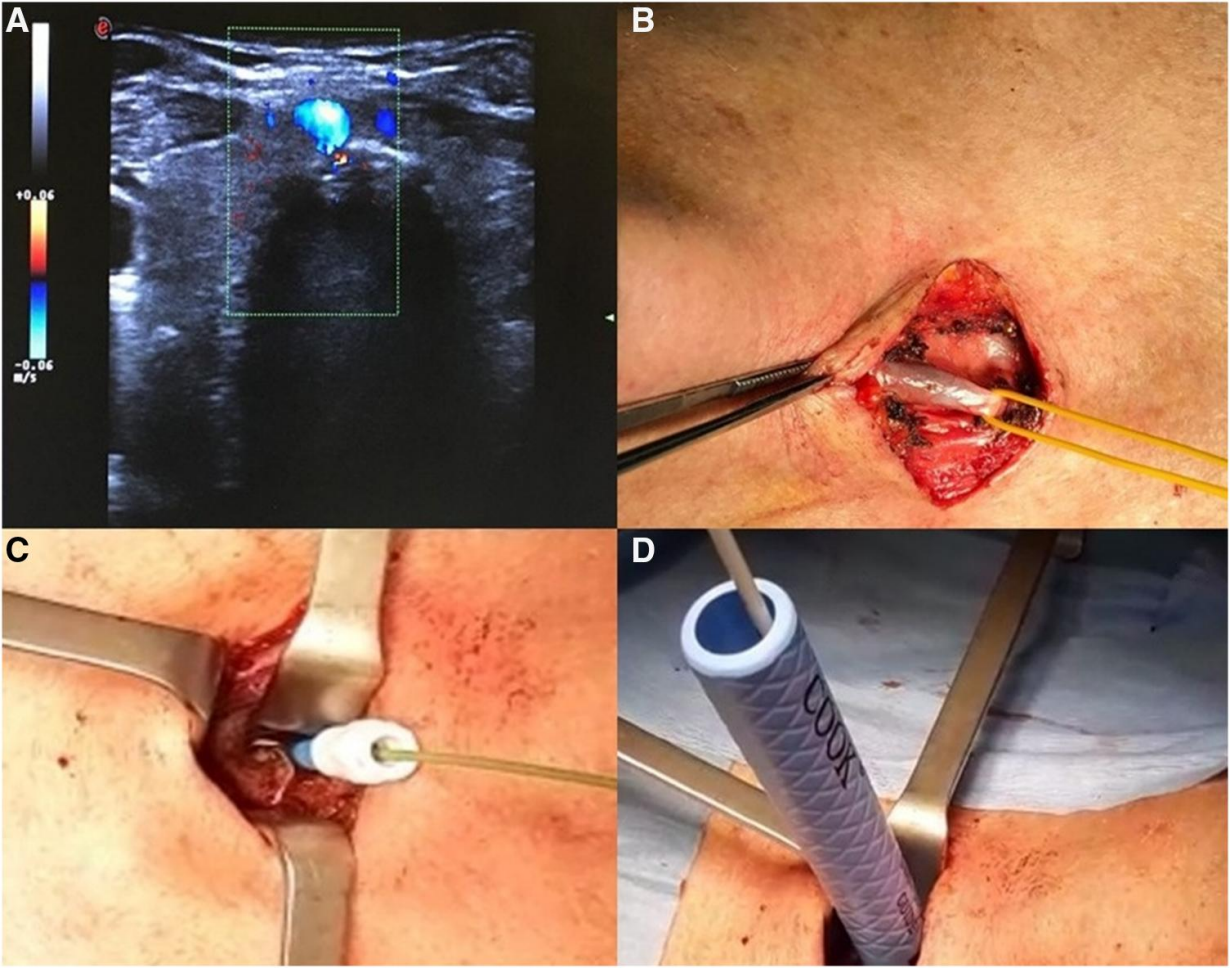
(A) Colour Doppler Ultrasound Shows an Abnormal Vascularization in the Neck Near the Jugular Notch of the Sternum; (B) Intraoperative View; and (C and D) Ciaglia Blue Rhino Tracheostomy

**Figure 2.**
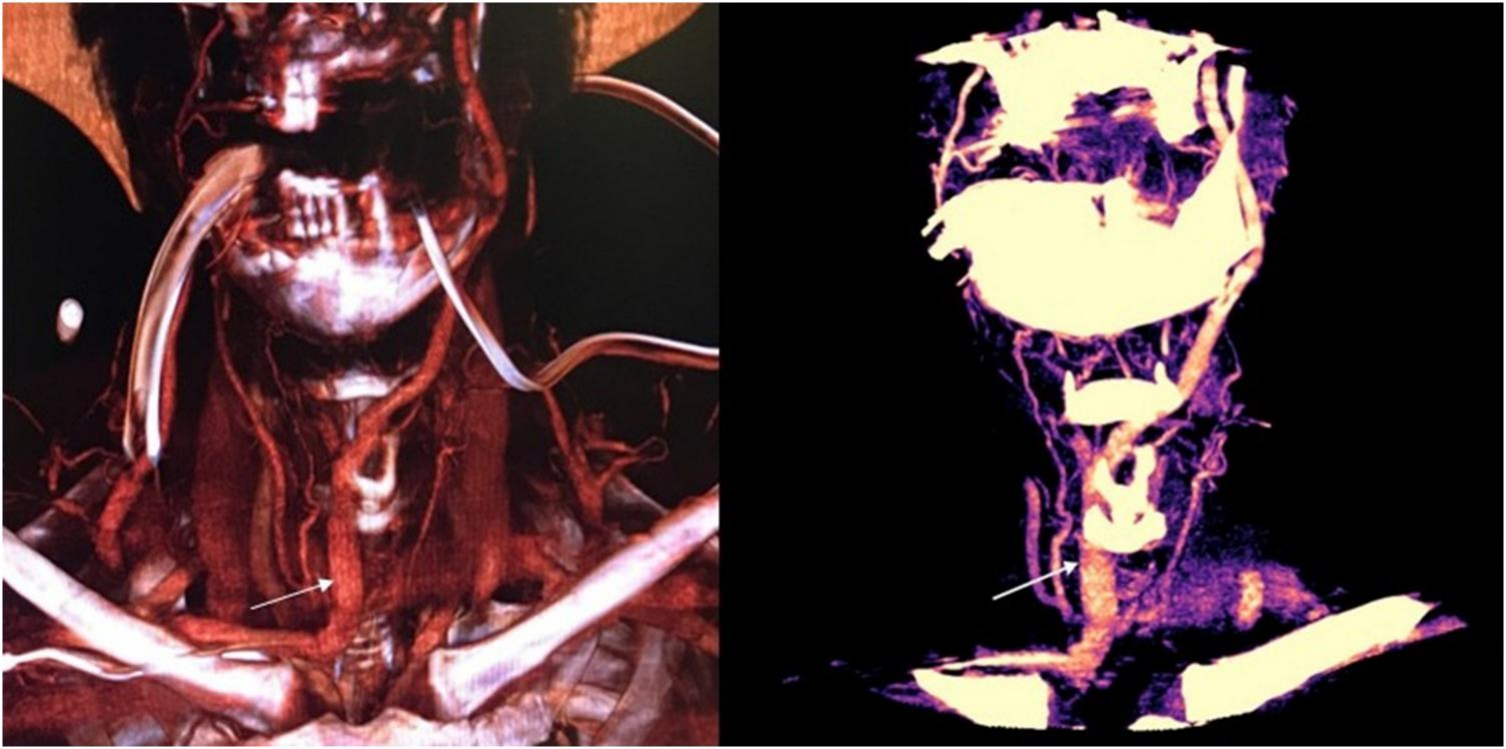
3D Volume Rendering Technique (VRT) and Para-Coronal Oblique Multiplanar Reformatting (MPR) Images Displayed an Ectatic Venous Collector That Drains from the Left Internal Jugular Vein into the Right Subclavian Vein. This Anomalous Vessel is Located Anterolaterally to the Trachea, Exposing the Patient to Bleeding Immediately During Tracheostomy or Late Due to the Continuous Contact of the Cannula on the Vessel

The clinical case refers to a 66-year-old male patient who showed multiple traumatic injuries (right temporal fracture, bilateral temporal contusions, left subdural haematoma at the skull level, twelfth rib fracture, somatic limitation of L4, right otorrhagia) and a Glasgow Coma Scale score of 5. After stabilization and intubation, he required prolonged ventilation support and a tracheostomy to proceed with weaning. A routine bedside preprocedural US screening revealed an abnormal paratracheal venous vessel (**Figure 1A**). Ultrasound is performed with colour-doppler technique; amplitude and depth of the US cone and signal intensity are set based on the anatomical characteristics of the patients. Our standard ultrasound technique is in cross-section (probe axis held perpendicular to the long axis of the trachea); additional views are also used to better delineate the structures of interest after identification in transverse images. During the ultrasound evaluation, the patient must be placed in a surgical (hyperextension of the head and neck; towel or pillow under the shoulders) and in a slight Trendelenburg position. It is necessary to maintain only minimal pressure with the probe to avoid darkening of the vascular structures caused by the compression of the probe itself. Following the US, the patient underwent a diagnostic angio-CT scan that confirmed an abnormal left jugular vein path. The vein passed in front of the tracheal axis and reached a maximum diameter of 1 cm at the level of the jugular dimple. Based on this finding, we decided to perform surgical tracheostomy using a hybrid technique (**Figure 1B-D**); written informed consent was obtained from the patient’s wife. The sternothyroid muscle flap was prepared and used to protect the anomalous vessel after its dissection and right displacement. To avoid contact between the edges of the surgical tracheal incision (H-shaped or T-shaped) and the vein, we opted for the “Ciaglia Blue Rhino” tracheostomy (**Figure 2**). Patient was discharged to a rehabilitation hospital and decannulated in the 15th postoperative day, returning to his usual life without sequelae.

## DISCUSSION

Percutaneous tracheostomy (PDT) is a commonly used procedure in intensive care. Several studies showed that an early PDT has been associated with a reduction in duration of mechanical ventilation, hospital stay and costs.^3^ Most frequent and fearful early complication is bleeding, due to unrecognized and unexpected anatomical variants of the vascular system or tracheal tube erosion of the adjacent vascular structure. Complications related to vascular anomalies could be minimized by a simple first-line neck ultrasound at the patient’s bed. This allows us to identify the calibre, course, and anomalies of the arterial or venous vessels and to establish the need for 3D reconstruction with angio-CT. Use of ultrasound before PDT shows several advantages: (1) identification of the thyroid gland and the vascular structures of the neck; (2) determination of the correct position of the tracheal hole; (3) choice a safe level for the insertion of the needle; and (4) detection of distance between skin and anterior tracheal wall. When an aberrant path has been identified preliminarily, the angio-CT of the epiaortic vessels and the neck, associated with 3D image reconstruction, optimizes the planning of the surgical strategy of tracheostomy; the use of an artificial intelligence platform may be advisable. Obviously, if advanced imaging resources are limited/non-existent within the same hospital or transport may entail significant logistical and clinical risks for the patient, it would be better to opt for bedside US and for tracheostomy directly. The choice of PDT must avoid the sharp blunt force injuries (tracheal cartilages) near the vessel. In our experience, the hybrid technique, performed centrally at the level of the II-III tracheal ring, precisely defined the cannula insertion area, reducing the likelihood of dislocation and late vascular injury due to the decubitus of the device on the vessel. This vascular anomaly truly posed a high risk in this case, and its relevance was more practical and not theoretical if one wants to avoid hemorrhagic complications. In conclusion, in case of vascular anomalies of the neck, it is necessary to develop a clear action plan to choose the most suitable tracheostomy approach, starting from the ultrasound and 3D CT angiography evaluation of the neck, if without risk for the patient, implementing internal procedures for patient safety.
